# Clinical Outcomes, Inflammatory Profile, Bacterial Co-Infections and Post-Acute Symptom Burden in Hospitalised COVID-19 Patients During the Omicron BA.5 Wave: A Single-Centre Cohort Study from Western Romania

**DOI:** 10.3390/microorganisms14051124

**Published:** 2026-05-15

**Authors:** Bogdan Adrian Manta, Diana-Maria Mateescu, Stela Iurciuc, Cris Virgiliu Precup, Camelia Corina Pescaru, Alina Andreea Tischer

**Affiliations:** 1 Division of Clinical Practical Skills, Faculty of Medicine, “Victor Babeș” University of Medicine and Pharmacy Timișoara, Eftimie Murgu Square 2, 300041 Timisoara, Romania; manta.bogdan@umft.ro; 2 Doctoral School, Department of General Medicine, “Victor Babeș” University of Medicine and Pharmacy Timișoara, Eftimie Murgu Square 2, 300041 Timisoara, Romania; diana.mateescu@umft.ro; 3 Cardiology Department, “Victor Babeș” University of Medicine and Pharmacy Timișoara, Eftimie Murgu Square 2, 300041 Timisoara, Romania; 4 Department of Life Sciences, “Vasile Goldis” Western University of Arad, 310414 Arad, Romania; 5 Center for Research and Innovation in Personalized Medicine of Respiratory Diseases (CRIPMRD), “Victor Babeș” Pneumology University Clinic, University of Medicine and Pharmacy Timișoara, Eftimie Murgu Square 2, 300041 Timisoara, Romania; pescaru.camelia@umft.ro; 6 Pulmonary Rehabilitation Center, Clinical Hospital of Infectious Diseases and Pulmonology, “Dr. Victor Babeș” Timișoara, Gheorghe Adam Street, No. 13, 300310 Timisoara, Romania; 7 Ear-Nose-Throat Department, “Victor Babes” University of Medicine and Pharmacy Timisoara, 300041 Timisoara, Romania; tischer.alina@umft.ro

**Keywords:** SARS-CoV-2, COVID-19, Omicron BA.5, vaccine effectiveness, reinfection, long COVID, post-acute sequelae, bloodstream infection, interleukin-6, Romania

## Abstract

Evidence on hospitalised COVID-19 patients during the Omicron BA.5 wave from Eastern European, vaccine-heterogeneous cohorts remains limited. We conducted a retrospective single-centre cohort study of 395 consecutive adults admitted with laboratory-confirmed COVID-19 to a tertiary infectious-diseases unit in western Romania between 1 July and 31 October 2022. Median age was 72 years (IQR 65–81); 33.2% were unvaccinated, 42.8% had documented prior SARS-CoV-2 infection, and 41.3% were obese. Multivariable logistic regression identified independent predictors of in-hospital mortality and post-acute symptom burden. In-hospital mortality was 15.7% (62/395). Vaccination was independently associated with lower mortality (adjusted odds ratio [aOR] 0.55, 95% CI 0.30–0.99; *p* = 0.048), as was each 1% increase in admission SpO_2_ (aOR 0.83, 95% CI 0.76–0.92; *p* < 0.001), whereas COPD independently increased mortality risk (aOR 2.42, 95% CI 1.15–5.10; *p* = 0.020). Interleukin-6 was the most discriminating admission biomarker for in-hospital mortality (AUROC 0.70). Bloodstream bacterial co-infection, detected in 22.5% of patients tested on clinical suspicion, was dominated by gut-derived organisms with case-fatality ≥30%. At discharge, 90.1% reported persistent symptoms, most commonly cognitive (24.6%). Prior SARS-CoV-2 infection independently predicted post-acute symptom burden (aOR 2.96, 95% CI 1.75–5.01; *p* < 0.001), with a specific cardiopulmonary signature. In this BA.5 cohort, vaccination remained protective; IL-6 was the most informative admission biomarker; bloodstream infections suggested gut translocation; and prior infection was an independent determinant of early post-acute symptom burden.

## 1. Introduction

By mid-2022, the global SARS-CoV-2 pandemic had transitioned from a wave dominated by immunologically naïve hosts to one in which most adults presenting to hospital had a complex pre-exposure history—any combination of prior infection, partial vaccination, full primary series, and one or more booster doses [1,2]. The BA.5 sublineage of Omicron emerged as the globally dominant variant during May–September 2022 and was characterised by greater transmissibility and substantial neutralisation escape from antibodies elicited by either ancestral-strain vaccines or earlier Omicron sublineages [3,4]. In Romania, BA.5 produced a distinctive sixth wave between July and September 2022, with a renewed peak in adult hospitalisations despite high cumulative population exposure [5].

This shift created an analytical problem that has been only partially addressed in the published literature. Many of the largest clinical descriptions of severe COVID-19 derive from the wild-type and Alpha/Delta waves, when the populations admitted were largely vaccine- and infection-naïve [6,7]. Studies from the Omicron era have often been embedded within mixed-variant cohorts, dominated by patients from Northern and Western European or North-American settings, and have rarely separated BA.5 hospitalisations as a discrete clinical entity [8,9]. Eastern European populations, in which vaccine uptake stalled at intermediate coverage and adenoviral-vector schedules (Janssen Ad26.COV2.S) coexist alongside two- and three-dose mRNA schedules, are under-represented in the hospital-based BA.5 literature [10].

Three clinically actionable questions remain incompletely resolved for this population. First, what is the residual benefit of prior vaccination—including a primary Janssen schedule administered 12–18 months earlier—against in-hospital death during a BA.5 admission, after adjustment for age and comorbidity? Second, which biomarkers measured at the time of admission still discriminate between survivors and non-survivors in an era when admission criteria themselves have shifted toward older and more comorbid patients? Third, in patients who survive to discharge, it is documented that prior SARS-CoV-2 infection is associated with a measurable difference in the burden or pattern of persistent symptoms—an important question given that reinfection is now the rule rather than the exception in any new wave [11,12].

This study makes three contributions that distinguish it from prior Omicron-era hospital cohort analyses. First, it provides one of the few BA.5-specific inpatient analyses from Eastern Europe—a geography under-represented in the international literature, characterised by heterogeneous vaccine schedules including the single-dose Ad26.COV2.S (Janssen) regimen. Second, it disentangles four discrete vaccination strata in a multivariable framework adjusted for age, comorbidity, and admission physiology, allowing direct comparison of a 12–18-month-old Janssen primary series with two- and three-dose mRNA schedules against a contemporaneous unvaccinated group. Third, it identifies documented prior SARS-CoV-2 infection—rather than vaccination status or comorbidity burden—as the primary independent predictor of early post-acute symptom burden at discharge, with a specific cardiopulmonary signature not previously characterised in a BA.5 Eastern European cohort. To address these aims, we analysed 395 consecutive adults hospitalised for laboratory-confirmed SARS-CoV-2 infection during the Romanian BA.5 wave at a tertiary infectious-diseases referral centre, capturing the heterogeneity of real-world practice in this geography—four vaccination strata, a broad comorbidity spectrum, and contemporaneous use of remdesivir and corticosteroids—while preserving the analytical depth required to quantify independent predictors of mortality, characterise bloodstream bacterial co-infection, and describe post-acute symptom clusters at discharge.

## 2. Materials and Methods

### 2.1. Study Design and Setting

This was a retrospective, single-centre, observational cohort study conducted at the Department of Infectious Diseases of a tertiary academic hospital in western Romania. The hospital is a regional referral centre for adult patients with severe community-acquired infections and provides dedicated COVID-19 inpatient and intensive care capacity throughout the pandemic. The study period was defined a priori as 1 July 2022 to 31 October 2022, corresponding to the locally documented Omicron BA.5 wave (the sixth pandemic wave in Romania, hereafter “Wave 6”) [5]. The reporting follows the STROBE statement for cohort studies [13].

### 2.2. Participants

We screened all adults (age ≥ 18 years) admitted during the study period with a positive SARS-CoV-2 reverse-transcription polymerase-chain-reaction (RT-PCR) test from a nasopharyngeal swab, performed locally on accredited platforms in accordance with World Health Organization case-definition criteria. We included patients fulfilling all of the following: (i) confirmed SARS-CoV-2 infection within 72 h of admission; (ii) clinical or radiological evidence of COVID-19 (lower respiratory symptoms, hypoxaemia, ground-glass opacities, or peripheral consolidations on chest imaging); and (iii) admission to the infectious diseases ward or to the COVID-19 intensive care unit. We excluded patients with incidental SARS-CoV-2 positivity in the absence of clinical or radiological disease and those who had spent <24 h in the institution. Variant assignment to BA.5 was epidemiological: during the study window, BA.5 accounted for >90% of sequenced isolates in Romania [5]. The final analytical cohort comprised 395 consecutive patients.

### 2.3. Variables and Data Sources

Data were extracted retrospectively by trained clinicians from the institutional electronic medical record using a structured case-record form and verified against the hospital laboratory information system. Demographic variables included age, sex, urban/rural residence, smoking status, and body mass index (BMI). Comorbidities were captured according to ICD-10 categories abstracted from the discharge summary and grouped a priori: cardiovascular (hypertension, atrial fibrillation, ischaemic heart disease, prior ischaemic stroke, and other cardiovascular conditions); chronic obstructive pulmonary disease (COPD); asthma; type 2 diabetes mellitus (T2DM); chronic kidney disease (CKD); hepatic; gastrointestinal; haematological; endocrine other than diabetes; and neurological. Vaccination status at admission was categorised as unvaccinated (NV), one dose of Ad26.COV2.S (Janssen, Leiden, The Netherlands), two doses of BNT162b2 (Pfizer–BioNTech, New York, NY, USA), or three doses of BNT162b2. Documented prior SARS-CoV-2 infection (“reinfection”) was defined as a previous laboratory-confirmed positive test recorded in regional or national surveillance databases at any point ≥90 days before the index admission [11].

Admission physiology comprised peripheral oxygen saturation on room air at first contact (SpO_2_), heart rate, and blood pressure. Admission laboratory variables were measured locally within 24 h of admission and included white blood cell count, erythrocyte sedimentation rate, C-reactive protein (CRP), interleukin-6 (IL-6), procalcitonin (PCT), ferritin, D-dimer, alanine aminotransferase, aspartate aminotransferase, urea, and creatinine. Laboratory analyses were performed using standardised automated platforms in the hospital’s accredited laboratory. SARS-CoV-2 infection was confirmed by reverse-transcription polymerase chain reaction (RT-PCR) using the cobas^®^ 6800 system (Roche Diagnostics, Mannheim, Germany). Blood cultures were processed using the BACT/ALERT^®^ 3D system (bioMérieux, Marcy-l’Étoile, France), with bacterial identification performed by matrix-assisted laser desorption/ionisation time-of-flight mass spectrometry (MALDI-TOF MS; Bruker Biotyper^®^, Bruker Daltonics, Bremen, Germany). Antimicrobial susceptibility testing was conducted using the VITEK^®^ 2 Compact system (bioMérieux, Marcy-l’Étoile, France), with interpretation according to European Committee on Antimicrobial Susceptibility Testing (EUCAST) criteria. Inflammatory markers, including interleukin-6, ferritin and procalcitonin, were measured using electrochemiluminescence immunoassays on cobas^®^ e601/e801 analysers (Roche Diagnostics, Mannheim, Germany). Haematological parameters were analysed using a Sysmex XN-1000 system (Sysmex Corporation, Kobe, Japan), and routine biochemical parameters were measured on a cobas c501 platform (Roche Diagnostics, Mannheim, Germany). Treatment variables included receipt of remdesivir, favipiravir, anakinra, systemic corticosteroids, supplemental oxygen, and any in-hospital antibiotic therapies. Bloodstream infection was defined as the growth of an organism considered clinically significant on at least one set of blood cultures drawn during admission; common skin-flora organisms growing in only one of multiple bottle sets were treated as contaminants and not counted. Blood cultures were obtained at the discretion of the treating physician, typically in patients with clinical suspicion of sepsis or clinical deterioration. Where available, timing of culture positivity was reviewed to distinguish early (community-associated) from late (hospital-acquired) infections; however, this distinction was not systematically recorded for all patients and is therefore not included in the primary analysis.

Outcomes were (i) in-hospital all-cause mortality, (ii) intensive care unit (ICU) admission at any point during the index hospitalisation, (iii) length of hospital stay, and (iv) post-acute symptom burden at the time of discharge, ascertained by a structured symptom checklist completed by the attending physician at discharge and grouped into six prespecified clusters: cardiopulmonary (cough, chest pain, palpitations); musculoskeletal (myalgia, arthralgia, headache); cognitive (memory loss, concentration difficulties, dizziness); respiratory/fatigue (fatigue, brain fog, dyspnoea); psychiatric (anxiety, depression, sleep disturbances); and gastrointestinal (abdominal pain, nausea, diarrhoea). Persistent symptoms at discharge are reported here as “post-acute symptom burden”; they do not satisfy the temporal criterion (≥3 months) of the WHO clinical case definition of post-COVID-19 condition [14] and are not interchangeable with that diagnosis (see Section 4).

### 2.4. Statistical Analysis

Continuous variables are summarised as median and interquartile range (IQR) when non-normally distributed (Shapiro–Wilk test) and otherwise as mean ± standard deviation; categorical variables as counts and percentages. Between-group comparisons used the Mann–Whitney U test for continuous variables and Pearson’s χ^2^ or Fisher’s exact test for categorical variables, as appropriate. Univariable and multivariable binary logistic regression were used to identify independent predictors of in-hospital mortality and of post-acute symptom burden. Variables for the multivariable mortality model (age, sex, BMI, vaccination status, COPD, prior cardiovascular disease, bloodstream infection, admission SpO_2_, prior SARS-CoV-2 infection) were prespecified on the basis of clinical relevance and prior literature; we did not perform stepwise selection. For the post-acute symptom model, adjustment variables were age, sex, BMI, vaccination status, COPD and prior cardiovascular disease, selected on the same basis. The discriminative ability of admission biomarkers for in-hospital mortality was quantified using the area under the receiver-operating-characteristic curve (AUROC). Two-sided *p*-values < 0.05 were considered statistically significant; no formal correction for multiple comparisons was applied, and exploratory tests are flagged as such. Analyses were conducted in Python 3.12 using pandas 2.2, statsmodels 0.14, scipy 1.13, and scikit-learn 1.5.

### 2.5. Ethics

Written informed consent for the use of anonymised clinical data for research purposes was obtained from all patients during their hospitalisation. The study was conducted in accordance with the Declaration of Helsinki [15] and was approved by the Ethics Committee of the “Victor Babeș” Clinical Hospital for Infectious Diseases and Pneumophthisiology, Timișoara, Romania (approval no. 4019/29 April 2026), which granted retrospective ethical clearance covering the full data-collection period (1 January 2020–31 December 2024). The present analysis uses the subset of patients admitted between 1 July and 31 October 2022. All data were anonymised prior to analysis.

## 3. Results

### 3.1. Cohort Characteristics

Between 1 July and 31 October 2022, 395 consecutive adults were admitted with confirmed COVID-19 and met the inclusion criteria. The cohort had a median age of 72 years (IQR 65–81; range 19.0–98.5), with the majority (75.4%) aged ≥65 years and 41.8% aged ≥75 years. Men accounted for 213/395 (53.9%) of admissions and rural residence for 187/395 (47.3%). Median BMI was 27.7 kg/m^2^ (IQR 24.6–30.9), and obesity (BMI ≥30 kg/m^2^) was present in 163 patients (41.3%). Comorbidity prevalence was high, in keeping with the older age structure: hypertension 79.0%, other cardiovascular disease 58.0%, prior ischaemic stroke 23.8%, ischaemic heart disease 19.0%, atrial fibrillation 17.2%, COPD 13.2%, T2DM 11.4%, and CKD 8.6% (Table 1). Active or former smoking was recorded in 33.9%.

At the time of admission, 131 patients (33.2%) were unvaccinated, 71 (18.0%) had received a single dose of Ad26.COV2.S (Janssen) ≥90 days before admission, 92 (23.3%) had completed a two-dose BNT162b2 primary series, and 101 (25.6%) had additionally received a BNT162b2 booster. One hundred and sixty-nine patients (42.8%) had documented prior SARS-CoV-2 infection. Symptom onset preceded admission by ≤5 days in 187 patients (47.3%) and by >5 days in 208 (52.7%); patients reported a median of 4 symptoms at admission (IQR 3–4). Median admission SpO_2_ on room air was 91% (IQR 89–93%); 130 patients (32.9%) presented with SpO_2_ < 90% and 10 (2.5%) with SpO_2_ < 85%.

### 3.2. Treatment, Complications and Clinical Course

Pharmacological treatment was largely homogeneous: 350 patients (88.6%) received remdesivir, 370 (93.7%) received systemic corticosteroids, and 285 (72.2%) received in-hospital antibiotic therapy at the discretion of the attending physician. Favipiravir was used in 12.2% (mainly during the first 4 weeks of the study window before remdesivir restocking) and anakinra in 4.1%. Supplemental oxygen was administered to 346 patients (87.6%) for a median of 3.5 days (IQR 0.6–7.8). Twenty-one patients (5.3%) required ICU admission, with a median ICU stay of 12 days (IQR 7–21). The median total length of hospital stay was 9 days (IQR 7–16): 8 days (IQR 6–13) in survivors and 13 days (IQR 7–22) in non-survivors. In-hospital all-cause mortality was 15.7% (62/395). Coded clinical complications recorded during admission included pulmonary complications (90.6%), cardiovascular complications (76.7%), hepatic complications (81.3%), renal complications (76.5%), sepsis (20.3%), septic shock (0.8%) and witnessed cardiopulmonary arrest (15.9%, mortality among these 55.6%).

### 3.3. Vaccination Status and In-Hospital Mortality

**Figure 1:**
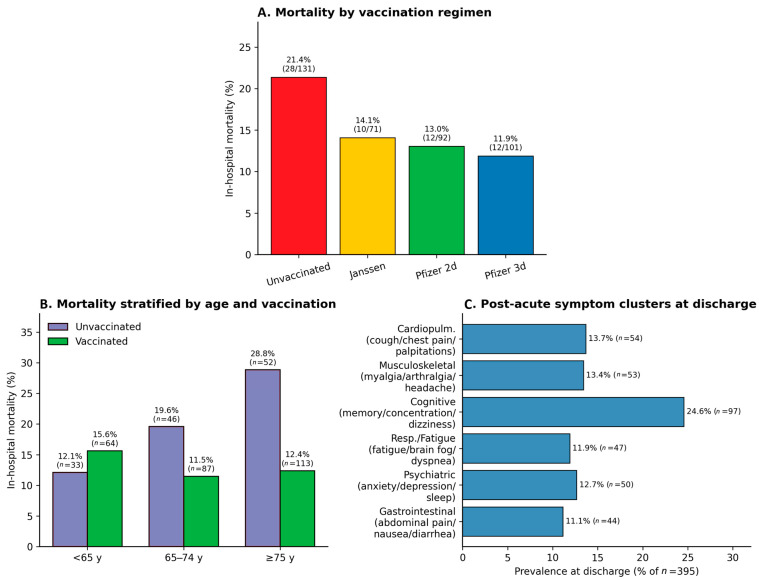
Cohort overview. (**A**) In-hospital mortality (%) by vaccination regimen; overall between-group *p* = 0.041 (Pearson’s χ^2^ test). (**B**) In-hospital mortality (%) stratified by age category (<65, 65–74, ≥75 years) and vaccination status (vaccinated vs. unvaccinated). (**C**) Prevalence (%) of post-acute symptom clusters reported at the time of discharge among survivors (*n* = 333). Clusters: cognitive (memory loss, concentration difficulties, dizziness), cardiopulmonary (cough, chest pain, palpitations), musculoskeletal (myalgia, arthralgia, headache), psychiatric (anxiety, depression, sleep disturbances), respiratory/fatigue (fatigue, brain fog, dyspnoea), gastrointestinal (abdominal pain, nausea, diarrhoea).

In-hospital mortality differed across vaccination strata (overall *p* = 0.041, χ^2^): 21.4% (28/131) in unvaccinated patients, 14.1% (10/71) after a single Janssen dose, 13.0% (12/92) after two doses of BNT162b2, and 11.9% (12/101) after three doses of BNT162b2 (Figure 1A). The pooled mortality among any vaccinated patients was 12.9% versus 21.4% in the unvaccinated, corresponding to an unadjusted odds ratio of 0.54 (95% CI 0.31–0.94; *p* = 0.030). The protective association was age-dependent (Figure 1B): in patients aged ≥75 years—the stratum with greatest absolute mortality—vaccination was associated with a >2-fold lower mortality (12.4% vs. 28.8%), whereas in patients <65 years the absolute numbers of events were small (4 deaths in 33 unvaccinated, 10 in 64 vaccinated) and no protective association was apparent. ICU admission was less frequent in vaccinated than unvaccinated patients (4.5% vs. 6.9%) but the difference did not reach statistical significance.

### 3.4. Admission Physiology, Biomarkers and Discrimination of Mortality

**Figure 2:**
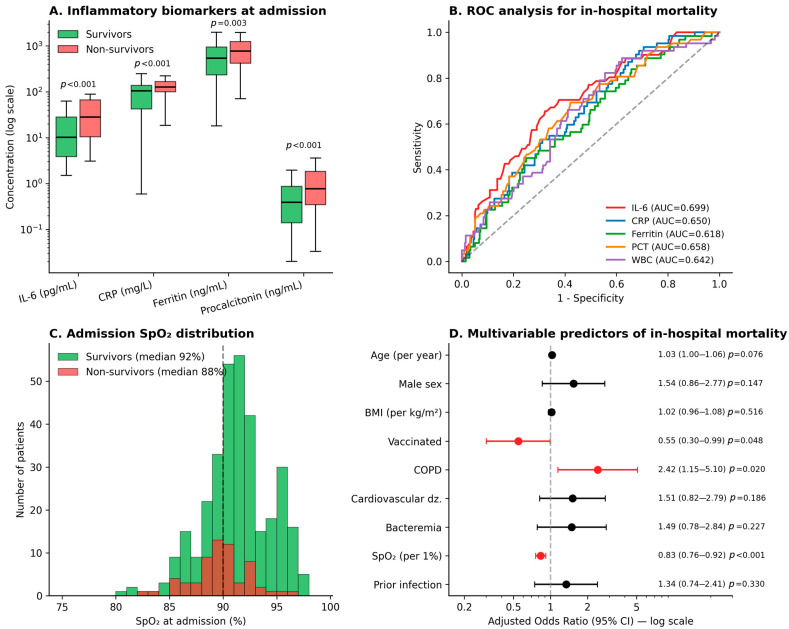
Admission inflammatory biomarkers and predictors of in-hospital mortality. (**A**) Tukey box-and-whisker plots of admission interleukin-6 (IL-6, pg/mL), C-reactive protein (CRP, mg/L), ferritin (ng/mL), and procalcitonin (PCT, ng/mL) in survivors (green) and non-survivors (red); y-axis on log_10_ scale. *p*-values from the Mann–Whitney U test; *p* < 0.001, *p* < 0.01. (**B**) Receiver-operating-characteristic (ROC) curves for individual admission biomarkers as predictors of in-hospital mortality; AUROC, area under the ROC curve; AUROC values are shown in the legend. (**C**) Distribution of admission peripheral oxygen saturation (SpO_2_, %) in survivors and non-survivors; *p* < 0.001 (Mann–Whitney U). (**D**) Multivariable logistic regression forest plot showing adjusted odds ratios (aOR) with 95% confidence intervals for in-hospital mortality (*n* = 395, 62 deaths). Predictors significant at *p* < 0.05 are shown in red, whereas non-significant predictors are shown in black.

Admission SpO_2_ was the strongest single physiological predictor of in-hospital death: median 88% (IQR 84–91) in non-survivors versus 92% (IQR 90–94) in survivors (*p* < 0.001) (Figure 2C). Admission inflammatory biomarkers were uniformly higher in non-survivors than in survivors: WBC count 13.5 versus 11.5 ×10^9^/L (*p* < 0.001), CRP 128 versus 105 mg/L (*p* < 0.001), IL-6 28.1 versus 10.2 pg/mL (*p* < 0.001), procalcitonin 0.77 versus 0.39 ng/mL (*p* < 0.001), and ferritin 776 versus 541 ng/mL (*p* = 0.003) (Figure 2A). D-dimer levels were marginally higher in non-survivors (0.81 vs. 0.72 µg/mL FEU; *p* = 0.057). Receiver-operating-characteristic analysis identified IL-6 as the best-performing single biomarker for in-hospital mortality (AUROC 0.70), followed by procalcitonin (0.66), CRP (0.65), WBC (0.64) and ferritin (0.62) (Figure 2B). A combined model incorporating all five biomarkers did not improve upon IL-6 alone (AUROC 0.67 vs. 0.70), most likely because of the strong inter-correlation among acute-phase reactants in this cohort.

In the prespecified multivariable logistic regression model for in-hospital mortality (Figure 2D, Table 2), independent predictors after adjustment for age, sex, BMI, COPD, prior cardiovascular disease, bloodstream infection, prior SARS-CoV-2 infection and admission SpO_2_ were: COPD (aOR 2.42, 95% CI 1.15–5.10; *p* = 0.020), vaccination (aOR 0.55, 95% CI 0.30–0.99; *p* = 0.048), and admission SpO_2_ per 1% increase (aOR 0.83, 95% CI 0.76–0.92; *p* < 0.001). Age showed a borderline association (aOR 1.03 per year; *p* = 0.076). Overall model fit was modest (Nagelkerke pseudo-R^2^ = 0.10); findings should therefore be considered hypothesis-generating rather than predictive.

### 3.5. Bloodstream Bacterial Co-Infection

**Figure 3:**
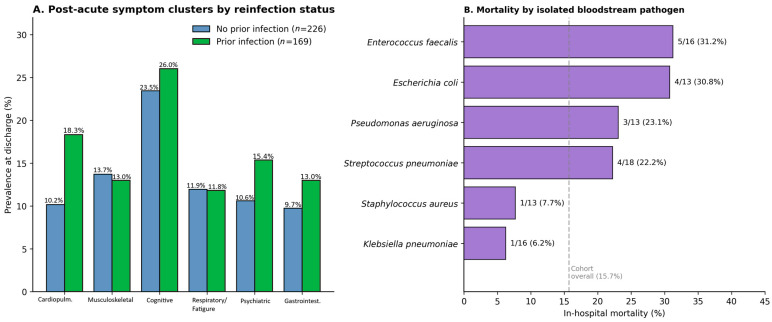
Reinfection and bacterial co-infection. (**A**) Prevalence (%) of six prespecified post-acute symptom clusters at the time of discharge, compared between survivors with (green; *n* = 169) and without (blue; *n* = 164) documented prior SARS-CoV-2 infection. Clusters: cognitive (memory loss, concentration difficulties, dizziness); cardiopulmonary (cough, chest pain, palpitations); musculoskeletal (myalgia, arthralgia, headache); psychiatric (anxiety, depression, sleep disturbances); respiratory/fatigue (fatigue, brain fog, dyspnoea); gastrointestinal (abdominal pain, nausea, diarrhoea). *p*-values from Pearson’s χ^2^ test; *p* < 0.05. (**B**) In-hospital mortality (%) among patients with positive blood cultures (*n* = 89), stratified by isolated pathogen species. The dashed grey line represents the cohort-overall mortality of 15.7%.

At least one positive blood culture during admission was documented in 89 of 395 patients (22.5%). The most frequently isolated species were *Streptococcus pneumoniae* (*n* = 18), *Klebsiella pneumoniae* (*n* = 16), *Enterococcus faecalis* (*n* = 16), *Staphylococcus aureus* (*n* = 13), *Escherichia coli* (*n* = 13) and *Pseudomonas aeruginosa* (*n* = 13). In-hospital mortality differed substantially across pathogen species (Figure 3B), with the highest case-fatality among patients with *E. faecalis* bacteraemia (5/16; 31.2%) and *E. coli* bacteraemia (4/13; 30.8%), and the lowest among patients with *K. pneumoniae* (1/16; 6.2%) and *S. aureus* (1/13; 7.7%) bacteraemia. Pooled mortality with any positive blood culture was 20.2% versus 14.4% with sterile cultures (unadjusted OR 1.51, 95% CI 0.82–2.77; *p* = 0.184). After adjustment for age, sex, BMI, vaccination, COPD, cardiovascular disease, prior infection and admission SpO_2_, bloodstream infection was not an independent predictor of in-hospital mortality (aOR 1.49; *p* = 0.227), although our analysis is underpowered for species-specific multivariable estimates. Antibiotic susceptibility data showed broadly the patterns expected for this geography (high penicillin susceptibility for *S. pneumoniae*, *S. aureus* and *E. faecalis*; uniform penicillin and macrolide resistance for the Gram-negative isolates), but the available data were aggregated at the species level and do not support a granular antibiogram presentation.

### 3.6. Post-Acute Symptom Burden at Discharge and Association with Prior Infection

At the time of discharge, 300 of 333 survivors (90.1%) reported at least one persistent symptom from the prespecified six-cluster checklist; the median number of symptoms at discharge was 1 (IQR 0–2). Cluster prevalences were: cognitive 24.6%, cardiopulmonary 13.7%, musculoskeletal 13.4%, psychiatric 12.7%, respiratory/fatigue 11.9% and gastrointestinal 11.1% (Figure 1C). The prevalence of any symptom at discharge was substantially higher in patients with documented prior SARS-CoV-2 infection than in those without (85.8% vs. 68.6%; χ^2^, *p* < 0.001). In a multivariable logistic regression model adjusted for age, sex, BMI, vaccination status, COPD and prior cardiovascular disease, documented prior infection was the only independent predictor of any post-acute symptom at discharge (aOR 2.96, 95% CI 1.75–5.01; *p* < 0.001); none of the other covariates reached significance.

Cluster-by-cluster analysis suggested a specific cardiopulmonary signature of reinfection (Figure 3A): the prevalence of cough/chest pain/palpitation symptoms at discharge was 18.3% in patients with prior SARS-CoV-2 infection versus 10.2% in those without (χ^2^, *p* = 0.029). Differences in the other clusters—including the dominant cognitive cluster—did not reach statistical significance, although a directional trend toward higher psychiatric symptoms (15.4% vs. 10.6%, *p* = 0.21) and gastrointestinal symptoms (13.0% vs. 9.7%, *p* = 0.39) in reinfected patients was observed.

## 4. Discussion

In this consecutive cohort of 395 adults hospitalised for laboratory-confirmed COVID-19 during the Romanian Omicron BA.5 wave, four findings stand out. First, vaccination delivered a measurable mortality benefit even in this elderly, multi-morbid, immunologically pre-exposed population: the adjusted odds of in-hospital death were almost halved among any vaccinated patient relative to the unvaccinated, and the absolute benefit was concentrated in the oldest stratum (Figure 1A,B, Table 2). Second, admission SpO_2_ retained the strongest physiological signal for in-hospital outcome, while interleukin-6 outperformed all other admission biomarkers individually with respect to mortality discrimination (Figure 2A–C). Third, COPD emerged as the most consistent comorbidity-level signal in the adjusted analysis, doubling the risk of in-hospital death (Figure 2D, Table 2). Fourth, documented prior SARS-CoV-2 infection was associated with a higher likelihood of carrying any post-acute symptom at the time of discharge, with a specific signal in the cardiopulmonary cluster (Figure 3A).

The observed predominance and high case-fatality of *Enterococcus faecalis* and *Escherichia coli* bloodstream infections suggest a potential gut-origin translocation mechanism, rather than a primary respiratory or device-associated source. In COVID-19, intestinal barrier disruption, systemic inflammation, and microvascular injury may promote translocation of enteric bacteria into the bloodstream. Extensive antibiotic exposure likely amplifies this process by depleting protective commensal microbiota and selecting for opportunistic overgrowth. Comparable gut-derived bacteraemia patterns have been reported in critically ill COVID-19 patients in other settings, supporting microbiota disruption as a shared driver of secondary infection risk.

The high rate of antibiotic use in our cohort (72.2%) reflects a common practice during the COVID-19 pandemic, particularly in older, comorbid populations with severe disease. However, accumulating evidence suggests that empirical antibiotic overuse in viral infections may contribute to microbiota disruption, selection of resistant organisms, and increased risk of secondary infections [16,17]. In our cohort, the substantial burden of bloodstream infections may be partially linked to this phenomenon, although causality cannot be established in this observational design. These findings highlight the importance of antimicrobial stewardship strategies even in the context of severe viral infections. The relatively high prevalence of bloodstream infection (22.5%) in our cohort should be interpreted in the context of selective blood culture testing, primarily performed in clinically deteriorating patients, which may overestimate the true incidence at the population level.

Our vaccine-related findings are consistent with the broader Omicron-era literature, including registry-based studies from the United States, the United Kingdom and Italy that documented persistent vaccine effectiveness against severe Omicron disease in the elderly even when neutralising antibody titres against BA.5 were substantially lower than against ancestral lineages [3,8,18,19,20]. We extend that literature in two ways: We separate four discrete vaccination strata, demonstrating a graded mortality association from unvaccinated through Janssen, two-dose Pfizer and three-dose Pfizer schedules; the pairwise adjusted comparisons did not individually reach significance because of limited per-stratum power, but the directionality and magnitude are coherent with prior data showing diminishing absolute risk with each additional dose [21]. We also document the persisting protection of a 12–18-month-old single Janssen dose—a point of practical importance in Eastern European populations where Janssen accounted for an unusually high share of the primary-vaccinated population during the 2021–2022 catch-up campaigns. The age-stratified pattern, with absolute benefit concentrated in the ≥75-year stratum, mirrors data from the UK Office for National Statistics and from Veneto, Italy [18,19].

The biomarker analysis reinforces the well-established role of IL-6 as a marker of severe COVID-19 [22,23,24] and adds nuance for the Omicron era specifically. The discrimination achieved by IL-6 alone (AUROC 0.70) was modestly better than CRP, ferritin or procalcitonin in our cohort, and a combined biomarker model produced little additional discriminative gain—likely reflecting the strong inter-correlation among acute-phase reactants. Procalcitonin discriminated mortality better than expected for a primarily viral infection (AUROC 0.66, with non-survivors having approximately twice the median value of survivors), almost certainly because elevated procalcitonin captures concurrent bacterial co-infection or secondary sepsis in a subset of severely ill patients. The simultaneous finding that bloodstream bacterial infection occurred in 22.5% of admissions but did not reach independent significance for mortality after adjustment suggests that procalcitonin captures the severity of any superimposed bacterial process more reliably than the binary fact of a positive blood culture in this setting [16,17].

The bloodstream-infection burden in our cohort is consistent with reports from Northern-European and Italian centres during late-Omicron waves, but the species distribution diverges in two respects [25,26,27]. Gram-negative organisms (*K. pneumoniae*, *E. coli*, and *P. aeruginosa*) accounted for almost half of all isolates, mirroring patterns described in nosocomial-acquisition studies from this geography. The disproportionate mortality among patients with *Enterococcus faecalis* and *E. coli* bloodstream infection (>30% case-fatality each) probably reflects gut translocation in critically ill or post-antibiotic-pressured patients rather than a direct virulence effect; the lower mortality with *Klebsiella pneumoniae* in our cohort, in contrast to several Italian series describing carbapenem-resistant Gram-negative organism colonisation and superinfection during COVID-19 hospitalisation [28], is reassuring and likely reflects the local antibiogram during the study period. We caution that with 13–18 isolates per species the species-specific mortality estimates have wide intrinsic uncertainty (Figure 3B).

Our most novel observation concerns the relationship between documented prior SARS-CoV-2 infection and post-acute symptom burden. In a cohort in which 90.1% of survivors had at least one persistent symptom at discharge, prior infection nearly tripled the adjusted odds of any persistent symptom (Table 2) and was specifically associated with a cardiopulmonary cluster (cough, chest pain, palpitations) in the cluster-stratified analysis (Figure 3A). The mechanistic substrate is plausible—repeated SARS-CoV-2 exposure causes incremental microvascular endothelial injury, autonomic dysregulation and persistent immune activation [29,30,31,32]—and is in line with the recent prospective cohort of Bowe and colleagues showing that reinfection adds substantial post-acute risk over and above the initial infection [11]. Our data complements that work in two important respects: it extends it to a Romanian, Eastern European cohort, and it identifies the cardiopulmonary cluster as the symptom domain most differentially associated with reinfection. We deliberately label this “post-acute symptom burden at discharge” rather than “long COVID” or “post-COVID-19 condition”, since assessment was at hospital discharge rather than at the ≥3-month time-point required by the WHO clinical case definition [14]; nonetheless, persistent symptoms at discharge are a strong clinical predictor of long-term post-acute sequelae [33,34], and therefore the observed reinfection signal is unlikely to dissipate at later follow-up.

### Limitations

This study has several limitations. First, the single-centre design and the timing constrained to the BA.5 wave limit generalisability to other settings, populations and variants. Second, variant attribution was epidemiological rather than sequence-confirmed at the individual-patient level; however, BA.5 represented the overwhelming majority of sequenced isolates in Romania during the study period [5]. Third, we relied on the routine medical record for comorbidity coding and on regional surveillance data for prior infection: under-ascertainment of comorbidities and especially of mild prior infections is plausible and would tend to bias our reinfection–symptom association toward the null, making our positive finding likely conservative rather than spurious. Fourth, the symptom checklist used at discharge captured only six prespecified clusters and did not quantify symptom severity or functional impact; we plan a prospective post-discharge follow-up to characterise the long-term trajectory. Fifth, our multivariable models had limited absolute event numbers (62 deaths), constraining the number of covariates we could include and preventing us from formally testing interactions between vaccination and reinfection. Sixth, antibiogram data were only available aggregated at the species level and we are therefore unable to present granular individual-isolate susceptibility patterns in this dataset.

Bloodstream infection may be subject to verification bias, as blood cultures were not systematically performed in all patients but rather in those with clinical suspicion of sepsis. Prior SARS-CoV-2 infection may be under-ascertained due to reliance on documented laboratory-confirmed cases, potentially leading to misclassification of reinfection status. The number of events relative to the number of variables included in multivariable models was modest, which may limit model stability and the precision of estimates.

## 5. Conclusions

In a 395-patient single-centre cohort representative of hospitalised COVID-19 in Eastern Europe during the Omicron BA.5 wave, vaccination remained an independent protective factor against in-hospital death (aOR 0.55), with the largest absolute benefit in patients aged ≥75 years; admission SpO_2_ and IL-6 were the strongest physiological and biochemical discriminators of mortality, respectively; bloodstream bacterial co-infection occurred in approximately one in four hospitalised patients and was dominated by gut-derived organisms; and documented prior SARS-CoV-2 infection was a robust independent predictor of persistent symptoms at discharge, with a specific cardiopulmonary signature. These findings support continued vaccination prioritisation in the elderly, integration of IL-6 and procalcitonin into routine admission risk-stratification, and explicit recognition of reinfection as a discrete clinical determinant of post-acute symptomatology. Prospective follow-up of this cohort beyond 3 months will be required to confirm whether the discharge-symptom signal translates into a durable post-COVID-19 condition phenotype.

## Figures and Tables

**Table 1:** Demographic, clinical, and laboratory characteristics of the 395 hospitalised patients with Omicron BA.5 COVID-19, overall and stratified by in-hospital outcome.

Characteristic	All Patients (*n* =395)	Survivors (*n* = 333)	Non-Survivors (*n* = 62)	*p*-Value
Age, years—median (IQR)	72 (65–81)	72 (64–80)	75 (68–83)	0.091
Male sex—*n* (%)	213 (53.9)	175 (52.6)	38 (61.3)	0.207
Rural residence—*n* (%)	187 (47.3)	156 (46.8)	31 (50.0)	0.648
BMI, kg/m^2^—median (IQR)	27.7 (24.6–30.9)	27.7 (24.5–30.7)	28.4 (25.6–31.7)	0.298
Obesity (BMI ≥ 30)—*n* (%)	163 (41.3)	133 (39.9)	30 (48.4)	0.221
Active or former smoker—*n* (%)	134 (33.9)	113 (33.9)	21 (33.9)	0.999
Vaccination status—*n* (%)				0.041
Unvaccinated	131 (33.2)	103 (30.9)	28 (45.2)	
Janssen, 1 dose	71 (18.0)	61 (18.3)	10 (16.1)	
Pfizer, 2 doses	92 (23.3)	80 (24.0)	12 (19.4)	
Pfizer, 3 doses	101 (25.6)	89 (26.7)	12 (19.4)	
Documented prior infection—*n* (%)	169 (42.8)	139 (41.7)	30 (48.4)	0.330
Comorbidities—*n* (%)				
Hypertension	312 (79.0)	266 (79.9)	46 (74.2)	0.239
Other cardiovascular disease	229 (58.0)	186 (55.9)	43 (69.4)	0.092
Ischaemic heart disease	75 (19.0)	62 (18.6)	13 (21.0)	0.797
Atrial fibrillation	68 (17.2)	60 (18.0)	8 (12.9)	0.426
Prior ischaemic stroke	94 (23.8)	78 (23.4)	16 (25.8)	0.571
COPD	52 (13.2)	39 (11.7)	13 (21.0)	0.076
Type 2 diabetes mellitus	45 (11.4)	38 (11.4)	7 (11.3)	0.978
Chronic kidney disease	34 (8.6)	31 (9.3)	3 (4.8)	0.258
Admission physiology				
SpO_2_, %—median (IQR)	91 (89–93)	92 (90–94)	88 (84–91)	<0.001
SpO_2_ < 90%—*n* (%)	130 (32.9)	91 (27.3)	39 (62.9)	<0.001
Admission laboratory—median (IQR)				
WBC, ×10^9^/L	11.8 (8.1–15.3)	11.5 (7.4–14.9)	13.5 (11.2–18.3)	<0.001
CRP, mg/L	109 (50–143)	105 (42–139)	128 (100–167)	<0.001
IL-6, pg/mL	10.7 (4.6–31.7)	10.2 (3.9–28.1)	28.1 (10.5–66.5)	<0.001
Ferritin, ng/mL	569 (267–1003)	541 (235–949)	776 (420–1245)	0.003
D-dimer, µg/mL FEU	0.75 (0.51–1.10)	0.72 (0.51–0.99)	0.81 (0.57–1.75)	0.057
Procalcitonin, ng/mL	0.40 (0.15–0.90)	0.39 (0.14–0.87)	0.77 (0.34–1.83)	<0.001
Creatinine, mg/dL	0.91 (0.63–1.38)	0.89 (0.62–1.35)	1.10 (0.71–1.56)	0.060

BMI, body mass index; COPD, chronic obstructive pulmonary disease; CRP, C-reactive protein; FEU, fibrinogen-equivalent units; IL-6, interleukin-6; IQR, interquartile range; SpO_2_, peripheral oxygen saturation; WBC, white blood cell count. Categorical comparisons by Pearson’s χ^2^ (or Fisher’s exact); continuous comparisons by Mann–Whitney U.

**Table 2:** Multivariable logistic regression model of in-hospital mortality (*n* = 395; 62 events). Variables prespecified on clinical grounds.

Variable	Univariable OR	Univariable 95% CI	Adjusted OR	Adjusted 95% CI (*p*)
Age (per year)	1.023	0.996–1.051	1.027	0.997–1.059 (*p* = 0.076)
Male sex	1.32	0.76–2.29	1.54	0.86–2.77 (*p* = 0.147)
BMI (per kg/m^2^)	1.01	0.96–1.06	1.02	0.96–1.08 (*p* = 0.516)
Vaccinated (any schedule)	0.54	0.31–0.94	0.55	0.30–0.99 (*p* = 0.048)
COPD	2.00	1.00–4.01	2.42	1.15–5.10 (*p* = 0.020)
Other cardiovascular disease	1.64	0.92–2.91	1.51	0.82–2.79 (*p* = 0.186)
Bloodstream infection	1.51	0.82–2.77	1.49	0.78–2.84 (*p* = 0.227)
SpO_2_ (per 1% increase)	0.84	0.76–0.92	0.83	0.76–0.92 (*p* < 0.001)
Documented prior infection	1.31	0.76–2.25	1.34	0.74–2.41 (*p* = 0.330)

BMI, body mass index; CI, confidence interval; COPD, chronic obstructive pulmonary disease; OR, odds ratio; SpO_2_, peripheral oxygen saturation.

## Data Availability

The original contributions presented in this study are included in the article. Further inquiries can be directed to the corresponding authors.

## Abbreviations

The following abbreviations are used in this manuscript:

aOR	Adjusted odds ratio
AUROC	Area under the receiver-operating-characteristic curve
BA.5	Omicron sublineage BA.5
BMI	Body mass index
CKD	Chronic kidney disease
COPD	Chronic obstructive pulmonary disease
CRP	C-reactive protein
FEU	Fibrinogen-equivalent units
ICU	Intensive care unit
IL-6	Interleukin-6
IQR	Interquartile range
NV	Not vaccinated
PCT	Procalcitonin
RT-PCR	Reverse-transcription polymerase chain reaction
SARS-CoV-2	Severe acute respiratory syndrome coronavirus 2
SpO_2_	Peripheral oxygen saturation
T2DM	Type 2 diabetes mellitus
WBC	White blood cell count
